# Universal ligation-detection-reaction microarray applied for compost microbes

**DOI:** 10.1186/1471-2180-8-237

**Published:** 2008-12-30

**Authors:** Jenni Hultman, Jarmo Ritari, Martin Romantschuk, Lars Paulin, Petri Auvinen

**Affiliations:** 1 Institute of Biotechnology, University of Helsinki, Viikinkaari 4, 00014 Helsinki, Finland; 2 Department of Ecological and Environmental Sciences, University of Helsinki, Niemenkatu 73, 15140 Lahti, Finland

## Abstract

**Background:**

Composting is one of the methods utilised in recycling organic communal waste. The composting process is dependent on aerobic microbial activity and proceeds through a succession of different phases each dominated by certain microorganisms. In this study, a ligation-detection-reaction (LDR) based microarray method was adapted for species-level detection of compost microbes characteristic of each stage of the composting process. LDR utilises the specificity of the ligase enzyme to covalently join two adjacently hybridised probes. A zip-oligo is attached to the 3'-end of one probe and fluorescent label to the 5'-end of the other probe. Upon ligation, the probes are combined in the same molecule and can be detected in a specific location on a universal microarray with complementary zip-oligos enabling equivalent hybridisation conditions for all probes. The method was applied to samples from Nordic composting facilities after testing and optimisation with fungal pure cultures and environmental clones.

**Results:**

Probes targeted for fungi were able to detect 0.1 fmol of target ribosomal PCR product in an artificial reaction mixture containing 100 ng competing fungal ribosomal internal transcribed spacer (ITS) area or herring sperm DNA. The detection level was therefore approximately 0.04% of total DNA. Clone libraries were constructed from eight compost samples. The LDR microarray results were in concordance with the clone library sequencing results. In addition a control probe was used to monitor the per-spot hybridisation efficiency on the array.

**Conclusion:**

This study demonstrates that the LDR microarray method is capable of sensitive and accurate species-level detection from a complex microbial community. The method can detect key species from compost samples, making it a basis for a tool for compost process monitoring in industrial facilities.

## Background

Composting is one of the principal methods to treat separately collected biodegradable waste. In composting, organic material is aerobically decomposed into humus-like material by bacteria, fungi and, to a lesser extent, other larger organisms [1]. To understand the composting process and the ecological processes of composting, the microbes present in the process need to be tracked. The compost microbiota have been characterised with a variety of molecular and cultivation based methods in both laboratory and full-scale processes (e.g. [2-6]. In order to follow the industrial composting process and to confirm the hygienisation of the compost, the microbiology needs to be understood and followed. Several approaches has been used to resolve compost microbiology, such as single-stranded conformational polymorphism (SSCP) [7], automated rRNA intergenic spacer analysis (ARISA) [5] denaturing gradient gel electrophoresis (DGGE) [3,4] and cloning and sequencing (Hultman *et al*. unpublished, Partanen *et al. *unpublished). Common to the technologies mentioned above is that they are rather time consuming and not suitable for routine determination of compost microbiota.

DNA microarrays have been widely used in studying gene expression for over 15 years. In addition, they present a promising approach for large scale microbial community analysis due to high-throughput at relatively low costs in contrast to methods relying on sequencing and culturing. Lately, the oligucleotide microarrays have been utilised in microbial species detection from various environments such as landfill methanotrophs [8,9], sulphate reducers [10] acidophiles [11] and composts [12]. The problems of oligonucleotide microarrays are largely due to the low hybridisation specificity which are not easily improved since increasing the length of the probes decreases specificity (reviewed in [13]). Also, the detection limit has been regularly reported to be around a 1% fraction of the total DNA [12,10]. Recently, detection levels of 0.1% have been reported [14,15] but these studies are not in every case applicable in environmental microbiology. High-throughput phylogenetic arrays can provide a fingerprint of the community under analysis, but are less optimal for species-level detection as the discriminatory power of oligonucleotide probes might not be good enough for distinguishing all the closely related species [8]. In this type of array design, the probes typically detect multiple targets requiring many probes per single target to make species-level detection possible [14,16]. Another type of microarrays widely used are the functional microarray by which the physiological status and functional activities of microbial communities can be analysed [17]. An example of the functional array is the GeoChip by He and coworkers [18] that contains probes for over 10 000 genes essential to various biogeochemical cycles.

Compost is an example of a microbial community in which the presence of certain key species is an important indicator of the functional status of the system. Detecting closely related species reliably from a complex sample requires high specificity and sensitivity in high-throughput format, making microarrays a promising platform for this kind of task. Enzymatic ligation based methods in microarray format have been used successfully in detection of environmental microbes at genus level [19-21]. The reaction is performed separately from the array hybridisation enabling the use of address oligos (other terms are tag or zip) to equalize probe hybridisation conditions. The enzymatic ligation step is the main source of specificity making it easier to design probes for highly similar targets.

**Figure 1:**
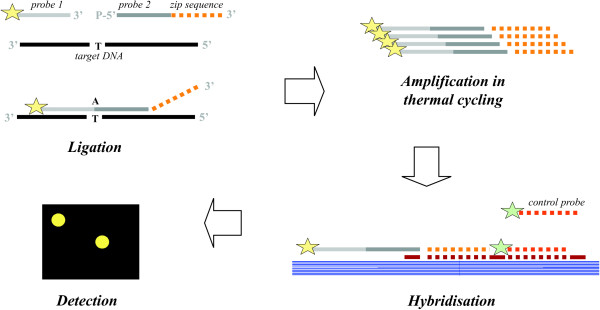
**Principle of LDR**. A schematic picture of the ligation detection reaction (LDR) [19] and hybridisation to the microarray by the zip-code sequences [25].

The principle of detecting specific DNA templates by enzymatic ligation was developed to overcome the limitations of oligomeric hybridisation probes in distinguishing single base mutations associated with genetic diseases [22,23]. The method relies on the high selectivity of a ligase which requires perfect complementarity of dsDNA structure to successfully catalyze the covalent joining of two adjacently hybridised probes. The probes constitute a target-specific probe pair, which becomes detectable only if the probes are linked together. The so called discriminating probe is designed such that the 3'-end matches the target at a unique position which contains a nucleotide that distinguishes the target from other species. The common probe is designed to hybridise adjacent to the discriminating probe enabling ligation if an appropriate target is present in the reaction mixture. Ligation products can be linearly or exponentially amplified in thermal cycling using thermostabile ligase [23], or in PCR following ligation [24]. In the universal microarray approach (Fig. 1), the common probe has a 3'-tag sequence (cZip code) which directs it to the right address on the array, while the discriminating probe is fluorescently labelled [25,19]. The advantages of the universal array lie in the uniform hybridisation conditions of all zip sequences, and in flexibility as the same array platform can be used with multiple ligation probe sets. The potential for high specificity and sensitivity makes ligation based detection techniques a promising tool not only for mutation screening but also for characterising complex microbial communities [19,21,26].

In this study, the LDR method was adapted to study microbes in compost samples. The focus was on the fungal communities of the composting process. The composition of communities correlates with efficiency of composting measured by various physical and chemical parameters (Hultman *et al. *unpublished, Partanen *et al. *unpublished). The aim of this study was to modify the LDR method into a more robust direction. In addition, the sensitivity and specificity of the probes and the array tests with closely similar fungal pure cultures and spiked clones in known concentrations were further evaluated. To overcome the problem of determining the desired signal level for positive hybridisation, the use of a control probe was tested in each printed spot. Finally, the method was used for eight compost samples taken from compost facilities in Finland, Norway and Sweden, from which clone libraries were constructed and sequenced to be used as a verification of the microarray hybridisation results.

## Methods

### Zip-code oligos

For printing, the zip sequence containing oligonucleotides were purchased from Oligomer Ltd (Helsinki, Finland). Each oligonucleotide carries a (dT)_9 _at the 5' end followed by the hybridisation control sequence (5'-TCAATGCACTGAGCCCGAGA-3'), poly(dT)_3_, a zip-code sequence and finally a poly(dT)_3_at the 3' end. The zip-code sequences and the hybridisation control probe sequence were selected from the Affymetrix GenFlex Tag Array (Affymetrix, Santa Clara, CA) data file, which was downloaded from the Affymetrix website.

### Probes

The probes were targeted to the ribosomal RNA of fungi. The studied area was the internal transcribed spacer (ITS) region of ribosomal RNA, comprising of the conserved 5.8S rRNA gene and variable flanking ITS1 and ITS2 regions. The rRNA sequences for all of the target and reference species were collected by sequencing [Hultman *et al. *unpublished, [27,28]] or from public databases. An alignment containing in total 11881 fungal ITS sequences was built with ARB software using ClustalW algorithm [29]. From the sequence alignment, unique discriminating nucleotides were identified for each target species and the LDR probes were designed such that the 3' position of each species specific probe matched the discriminating nucleotide of the target species. The common probes were located immediately after the discriminating probes according to the corresponding target sequences. The Tm's were set to 65°C calculating with NetPrimer software . The probes were searched with BLAST [30] against all public nucleotide sequence databases and discriminating probes were discarded from further use if an extensive 3' end similarity to non-target sequence was found. The discriminating probe was equipped with a Cy3 molecule in the 5' position. The common probes contained a phosphate in the 5' terminal position and a complementary zip-code (czip-code) in the 3' end. The complementary hybridisation control probe B3 (Table 1) was purchased with 5' Fam dye. The oligos were purchased from Oligomer Oy (Helsinki, Finland).

**Table 1:** Probe sequences, target organisms and complementary zip-code sequences

**Probe name**	**Target organism**	**Discriminating probe**
PenCtr (A26)	*Penicillium citrinum*	GCCTCGGCGGGCCCC
Pezizomycota (A29)	*various Penicillia and other*	GTCCGGTCCTCGAGCGTATGG
NP101 (A45)	*Environmental clone from compost*	ATCAAAGTGCTGCAGGGCT
PenCom (A50)	*Penicillium commune*	CCCCGTCCTCCGATCTCCG
IssOr (A52)	*Issatschenkia orientalis*	GCGGACGACGTGTAAAGAGC
CanEt1 (A53)	*Candida ethanolica*	ACGCTTGGGGTCTCCGAG
GalGe (A54)	*Galactomyces geotrichum*	ACAACACTATTCAACCTCAGATCA
TherLa (A55)	*Thermomyces lanuginosus*	CACTGTGAACGCTTTTGTGAA
CanEt2 (A56)	*Candida ethanolica*	CACGAGCGAACTAGAACAGG
GeoEme (A57)	*Geosmithia emersonii*	AGACCCTCGTGAACGCTGT
AspFum (A58)	*Aspergillus fumigatus*	AGACCCCAACATGAACGCTG
Emesp A(65)	*Emericella *sp, *Trichocomaceae *sp.	CCGGGGACCACTGAACTTC
AbsCor (A66)	*Absidia corymbifera*	AGGTCTTCTCTTAAGGTTCCTCAC
PichFerm2 (A67)	*Pichia fermentas*	TTCTTGCGCAAGCAGAGTTG
KlyMa1 (A83)	*Kluyveromyces marxianus*	GCTTAATTGCGCGGCCAG
KlyMa2 (A84)	*Kluyveromyces marxianus*	TCATCCTCTGCTATCAGTTTTCTA
CanRu (A85)	Environmental clone similar to *C. rugosa*	ACAATAATTCAACATTGTGTCAGAG
Pichferm (A86)	*Pichia fermentans *and *P. kluyveri*	TGAACGCACATTGCGCCC

**Probe name**	**Common probe**	**Zip code**

PenCtr (A26)	GCGCCCGCCGACGG	GTACTAGCATATCATCGACG
Pezizomycota (A29)	GGCTTTGTCACCCGCTCTGTAG	GTTCATCACGAGTGCGTAGA
NP101 (A45)	AGCGCGCCTCCGTGTAG	CGTACAGTAAGTATGATGCC
PenCom (A50)	GGGGACGGGCCCGAAAGG	TTAATTGACTTCGCTCCAGC
IssOr (A52)	GTCGGAGCTGCGACTCGC	AAATCAGCAAACGGGCTCCG
CanEt1 (A53)	CACTATGAGCTCGACCTCAGAT	GAATTGATAATCGCAGCCAC
GalGe (A54)	AGTAGGATTACCCGCTGAACTTAA	GATATAGGAATGGCGCATAC
TherLa (A55)	TGCGAGGATTGTCTGAGTGAC	CTCATCGGAAGGGCTCGTAA
CanEt2 (A56)	ACGCTTGGGGTCTCCGAG	ACAGATGGAAAGGCAGTTCT
GeoEme (A57)	CTTGAACAAAGGTTGCGGTCT	TTTGGTAGCTGAGTGCCCTA
AspFum (A58)	TTCTGAAAGTATGCAGTCTGAGTTA	TAACTGGTTTGACGCCACGC
Emesp A(65)	ATGCCTGAGAGTGATGCAGTC	CTTCTGTCAATATGGGTACG
AbsCor (A66)	AGTTATGTGCAATGTTGGGTCAC	TATTTCGAGATATGAGGCGC
PichFerm2 (A67)	AGAACAGGCTATGCCTTTTTCG	TTGATCGTAGATTCGTGAGC
KlyMa1 (A83)	TTCTTGATTCTCTGCTATCAGTTTT	CACTAATTCAGACGAAGCCG
KlyMa2 (A84)	TTTCTCATCCTAAACACAATGGAG	GACCCTATCAGACAGATGCA
CanRu (A85)	CAATAACATCTAAAACCGATCATC	CACGCATCAAGACAGTATCG
Pichferm (A86)	CATGGTATTCCATGGGGCAT	CAGCTCCTAAGACTTGGACA
B3 control		TCTCGGGCTCAGTGCATTGA

### Array and printing solution testing

The first microarrays were printed on four different glass types with eight printing solutions in order find the most suitable combination. Two identical subarrays were printed on each slide. The microarray slides used were Nexterion slide A, Nexterion slide A+, Nexterion slide E (Schott Nexterion, Mainz, Germany) and Corning Ultra GAPS (Corning Life Sciences, Amsterdam, Netherlands). Buffers used were 6 × SSC, 2 × Micro spotting solution (MSS; TeleChem International, CA, USA), 2 × Micro spotting plus (MSP, TeleChem International), 2 × Nexterion spot III (Schott), 2 × Nexterion spot (Schott), 1 × LEB I, 1 × LEB II and 1 × LEB III (Schott). Final concentration of the buffers was 1× with the exception of 6 × SSC which was printed in concentration 3×. The probe concentration in the printing buffer was 50 μM. Quality control to the different slide types was made by hybridising one subarray with labelled complementary hybridisation control and another subarray was stained with SYBR Green II RNA Gel Stain (Invitrogen, CA, USA). The slides were scanned with GenePix Autoloader 4200AL scanner (Molecular Devices, CA, USA) using wavelength 488 nm and quantified by GenePix (Molecular Devices).

The second slide batch was made on Nexterion A-slides with three printing buffers (MSP, MSS and Nexterion 3) with two identical subarrays per slide. Final batch of slides was made on Nexterion Slide A MPX 16 with MSS-printing buffer since it appeared to be the best combination, giving good signal and spot quality. Sixteen identical subarrays were printed on the slide. All slides were printed in the Biomedicum Biochip Center, University of Helsinki, Finland, and after printing the slides were UV cross-linked with 1000 × 1000 μJ of 254 nm UV light (UV Stratalinker 2400, Stratagene, CA, USA).

#### Sample preparation

The samples used to test the LDR probes were PCR amplified from genomic DNA of strains kept in our culture collections or from environmental clone libraries. The pure culture DNA was extracted with commercial kit (MasterPure Yeast DNA Purification Kit, Epicentre, WI, USA). The fungal strains were grown on malt extract agar, DG18, or potato carrot agar. After 7 days growing at 25°C, all plates were examined macroscopically and microscopically to check for purity and correct identification. In case of contamination, strains were recultivated. Microscopic identification was based on colony morphology, i.e. their growth habit, structure, colour, mycelium and spores. The identification based on morphology was done at genus level, sometimes at species level. In addition, the ITS1- and ITS2-regions of all strains were sequenced to confirm the identification. Spores from well-grown and verified cultures on agar plates were collected in buffer solution and stored at -80°C. The PCR amplification was performed with primers Fun18f (Hultman *et al. *unpublished) and ITS4 [31] in 50-μl volume containing 1× Dynazyme buffer (Finnzymes, Espoo, Finland), dNTP solution (200 μM of each dATP, dCTP, dGTP and dTTP), 0.5 μM primers, 1 U DNA polymerase (DynazymeII, Finnzymes, Espoo, Finland) and varying amount of template DNA. The PCR reaction was carried out in a thermocycler (MJ Research, MA, USA) under the following conditions: denaturation of 5 min at 94°C, followed by 25 cycles of 94°C for 30 s, 50°C for 30 s and 72°C for 45 s, with a final extension of 10 min at 72°C. PCR products were purified with MultiScreen PCR_384_purification plates (Millipore, MA, USA).

#### Ligation

The LDR was carried out in a final volume of 20 μl containing 1× ligation buffer (TAQ ligase buffer, New England Biolabs, MA, USA), 30 mM tetramethylammonium chloride (TMAC), 250 fmol of each discriminating probe, 250 fmol of each common probe, 5 pmol of the complementary hybridisation control probe, and a variable amount of purified PCR products. After the reaction mixture was preheated for 2 min at 94°C and centrifuged for 1 min, 4 U of Taq DNA ligase (New England Biolabs) was added. The LDR was cycled for 40 rounds at 94°C for 30 s and at 64°C for 4 min in a thermocycler (MJ Research)

In the testing phase of the method the success of the ligation was visualised with polyacrylamide gel electrophoresis (PAGE, MINI-protean 3 cell, Bio-Rad, CA, USA). Ligation products were denatured and 20 ul was loaded on 18% PAGE gel with 10 ul of formamide and 3 ul of 10× gel loading dye. The gel was run at 200V for 1 h, stained with SYBR Green II RNA Gel Stain and visualised with a Dark Reader instrument (Clare Chemical research, CO, USA).

### Template and probe testing

Ligation sensitivity was validated by titrating the PCR-products serving as templates for ligation to 10 fmol, 1 fmol, 0.1 fmol and 0.01 fmol. In addition, spiking experiments were done with the same template volumes but either 100 ng of Herring sperm DNA or genomic DNA from various *Penicillium *species used as a background DNA. Ligation and hybridisation were performed as described above and the titration experiments were conducted as well. The probe specificity was tested in hybridisation with all of the 17 probe pairs (Table 1.) and one of the templates for all of the matching templates individually.

### Hybridisation, washing and scanning

In a 1.5 ml microcentrifuge tube, the LDR mix (20 μl) was diluted to obtain 40 μl of hybridization mixture containing 5× SSC and 0.1 mg/ml herring sperm DNA. After heating the mix to 94°C for 2 min and chilling on ice, ligation control probe was added and the mix was applied onto the slide according to slide manufacturer's instructions. Hybridization was carried out in the dark at 50°C for two hours, in a temperature controlled hybridisation oven. After hybridisation, the microarray was washed for 3 × 15 min in 0,1× SSC, 0.1% SDS and briefly with water. Finally, the slide was dried in a table centrifuge. The fluorescent signal was detected at 5 um resolution using a GenePix Autoloader 4200AL laser scanning system with green laser for Cy3 dye (ex 543 nm/em 570 nm, LDR-probe) and blue laser for 6-FAM (ex/em 488, control probe B3). Both the laser and the photomultiplier (PMT) tube power were set at 100%. GenePix program version 6.0 was used to quantitate the fluorescent signal from each spot.

### Microarray data-analysis

The data were analysed using R statistical environment v. 2.6.2 [32]. Package Marray v. 1.16.0 [33] from the Bioconductor project [34] was used to manage the microarray data in R. The negative values resulting from subtracting local background values from each spot in a subarray were substituted with median value calculated from the spots without LDR probe in the subarray. Missing spots were identified as having values more than 2 standard deviations below median. LDR signal in a spot was substituted with median value of spots without LDR probe if the corresponding B3 control probe value was identified as missing. The log ratio of LDR and B3 signals was calculated for each spot. Spots with no LDR probe were used as a background set against which spot replicates of each probe were compared. Spots with ratios over 2.5 SD of background median were identified as positive. The R-scripts are available from authors upon request.

### Validation with compost samples

DNA was extracted with Fast prep for soil kit (Irvine, CA, USA) from ten compost samples (Table 2.) from full-scale facilities in Norway, Sweden and in Finland [35,36]. The samples, used here as reference samples, were produced and mainly analysed as part of a Nordic composting project [35,36]. The samples NK05 to NK10 were from the research reactor. The reactor experiments (200-liter) were temperature and oxygen controlled and during the run the process was allowed to self-heat to 37°C but thereafter cooled to keep the temperature below 40°C until the pH was above 6 when the temperature was increased to 55°C. By controlling the temperature, the acidic phase was rapidly overcome as the efficient decomposition started and the effect of the inhibitory acids at low pH was avoided [37]. The oxygen concentration was kept at 16%. Samples from NK05 to NK07 were sequential during the composting process where the temperature was set to 55°C. Clone libraries were constructed from samples NK06 and NK07. Samples NK08, NK09 and NK10 were from a process where the temperature was gradually increased starting on day 9 to reach 70°C. The sample NK08 was taken on day 0 from the waste material to be composted. Samples NK12 and NK14 were from two batches in a Norwegian facility with agitated beds. Sample NK12 was from with a batch where the temperature was about 60°C most of the time, but below 50°C during the last week before sampling, and sample NK14 was from a batch where the temperature was 60–70°C. Sample NK19, taken from a composting plant in Finland, was from a compost tunnel with floor area of 110 m^2 ^and filled with 140 t of biowaste and 40 t of structure material. At the time of sampling, reduced aeration was used in order to prevent the drying of the compost. Sampling was performed as described by Sundberg et al. [35].

**Table 2:** Clone library information.

**Sample**	**Clones**	**Phylotypes**	**Chao**	**ACE**	**Simpson**
NK05 – research reactor	-	-			
NK06 – research reactor	69	14	30.0	37.0	4.9
NK07 – research reactor	17	5	6.0	6.4	2.4
NK08 – research reactor	-	-			
NK09 – research reactor	91	12	24.3	17.1	1.7
NK10 – research reactor	82	10	11.0	14.2	2.8
NK12 – full-scale (IVAR)	69	18	34.7	40.6	5.9
NK14A – full-scale (IVAR)	97	11	29.0	24.3	2.4
NK14B – full-scale (IVAR)	89	9	9.0	27.5	2.6
NK19 – full-scale (YTV)	74	3	3.5	4.0	1.1

The predicted richness estimates were done based on Chao [39] and Chao & Lee [ACE, [40]], Simpsons reciprocal indices by Simpson [41].

The PCR amplification was performed in triplicate with primers Fun18f (Hultman *et al. *unpublished) and ITS4 [28] in 50-μl volume containing 1× Phusion GC buffer (Finnzymes, Espoo, Finland), dNTP solution (200 μM of each dATP, dCTP, dGTP and dTTP), 0.5 μM primers, 5% DMSO, 1U DNA polymerase (Phusion, Finnzymes, Espoo, Finland) and varying amount of template DNA. The PCR reaction was carried out in a thermocycler (MJ Research) under the following conditions: denaturation of 30 s at 98°C, followed by 25 cycles of 98°C for 10 s, 55°C for 30 s and 72°C for 30 s, with a final extension of 5 min at 72°C. PCR products were purified and replicates pooled using MultiScreen PCR_384_purification plates (Millipore, MA, USA). For the ligation reaction 10 ng of genomic DNA and 4 ng and 20 ng of purified PCR product were used as a template with 250 fmol of probes (Table 1). The data-analysis was done as describe above. The ligation and hybridisation with 20 ng of PCR products was done in three replicates. When two out of the three replicates gave positive signal, the probe was considered positive.

### Cloning and sequencing of samples

The eight compost samples used in ligation were also sequenced in order to compare the results from these two methods. A DNA-library was constructed and clones from each library were sequenced as in Hultman *et al*. (unpublished). Ninety-six clones were sequenced from each library expect from sample NK07, from which the number was 35 clones. The sequences that passed the Phred 20 quality test [38,39] and were joined as phylotypes with 99% similarity and aligned to Genbank with BLAST algorithm [30]. The multiple alignment for the sequences was done with Muscle [40] provided by CSC (The Finnish IT center for science) and the phylogenetic analysis with Phylip package [41]. The phylogenetic tree was constructed by the neighbor-joining method [42] and the sequences from two Chytridiomycota, *Orpinomyces *sp. (AJ864475) and *Anaeromyces *sp. (AY429667) were used as outgroups. Data sets were bootstrapped with 1000 random replicates. The random seed number was 111. The tree was illustrated by using a NJ-Blot program [43]. Chao1 [44], ACE [45] and Simpsons reciprocal index [46] were used for the coverage and richness estimations of the libraries.

All chemicals and solvents were purchased from Sigma-Aldrich (Finland) and used without further purification. Custom synthesized oligonucleotides were purchased from Oligomer Oy (Helsinki, Finland). The sequences have been submitted to EMBL nucleotide sequence database with accession numbers FM177644–FM177696.

## Results

### Sensitivity

**Figure 2:**
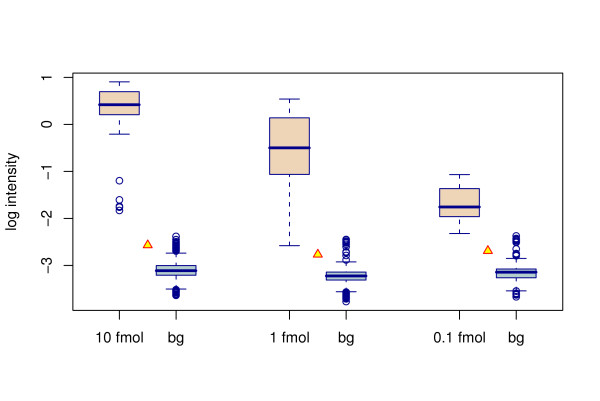
**Probe sensitivity**. Boxplots showing the signal distribution of probes detecting different concentrations of template. "bg" is the background distribution in the same subarray as a given template. Yellow triangles denote the 2.5 SD detection limit above the background median. The false positives above the detection limit are from cZip number 17 which was not used in any of the probes.

**Figure 3:**
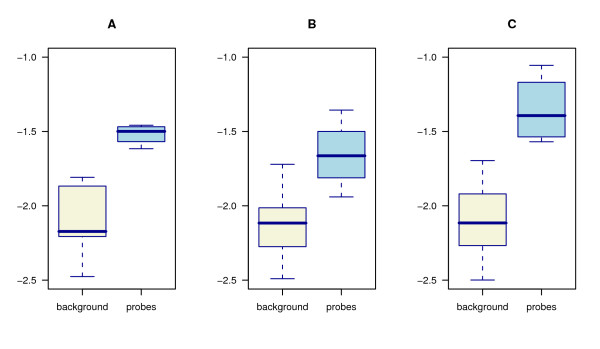
**Effect of ligation cycle number on the probe signals**. The distributions of ligation probe signals after A) 40 cycles B) 80 cycles and C) 120 cycles of ligation.

The absolute detection limit and relative sensitivity of the probes was evaluated using artificial mixes of PCR fragments from fungal pure cultures and clone libraries of environmental samples. In a mixture containing 100 ng of herring sperm DNA or *Penicillia *DNA, 0.1 fmol of PCR product was seen to be sufficient to give positive signal (Fig. 2). No differences in results could be observed whether the background DNA was herring or *Penicillium*. The detected amount, 0.1 fmol of on average 700 bp PCR product, corresponds to ca. 0.04% sensitivity level. To help normalising zip-oligo hybridisation signals over different spots and arrays, a control probe sequence was included in each zip code oligo (Fig. 1). The thermodynamic melting temperature properties of the control probe were similar to the actual zip sequences. The control probe was utilized as an indicator of per-spot zip sequence hybridisation variability and setting limits for detection. Increase in number of ligation reaction cycles was not found to affect to the sensitivity (Fig. 3).

### Specificity

**Figure 4:**
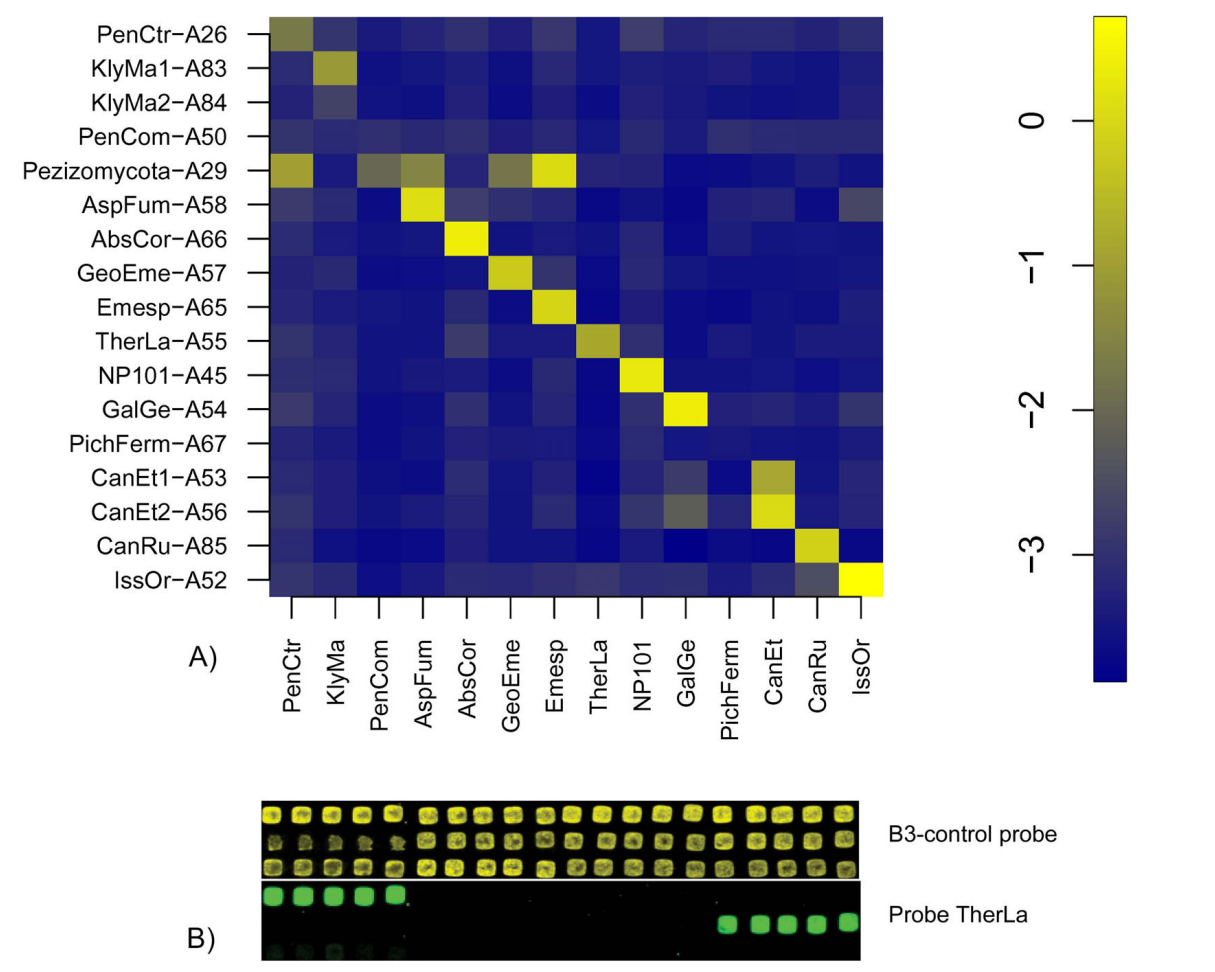
**Probe specificity**. a. The intensity values of all the probes against individual templates. On the Y-axis, the set of probes present in each reaction. On the X-axis, the template present in a given reaction. The colour coded values are intensity log ratios of LDR/B3. Highest values are expected to be in the diagonal. Three probes, PenCom-A50, KlyMa2-A84 and PichFerm-A67, do not detect any template. Probe AspFum-A58 is slightly non-specific to *I. orientalis *template. Pezizomycota-A29 is group-specific. b. Scanned images of an example slide with hybridisations of the complementary control probe and two probes specific for *Thermomyces lanuginosus *phylotype (TherLa in Fig. 4a) The control probe can be seen to hybridise to all of the spots and the specific probe for the corresponding zip-code sequences.

To estimate the specificity of the probes, ligation reactions each containing a different template and all of the probes were prepared and hybridised to different MPX subarrays. Of the fourteen phylotype-specific probes tested, three did not give sufficient signal to be considered functional: PenCom, PichFerm and KlyMa2 (Fig. 4). One probe was observed to give nonspecific signal: AspFum probe detected *Issatschenkia orientalis *(IssOr) template, although the level of signal was barely over the threshold. In addition, probe CanEt2 seemed to detect GalGe template weakly, but this was expected because CanEt probes were designed to detect their specific template only as a pair. The rest of the 14 phylotype specific probes were specific to corresponding template despite the taxonomical relatedness. The probe for Pezizomycota-fungi proved to be group specific as well.

### Testing with environmental samples

To determine how the detection functioned when applied to complex environmental samples, the probes were tested with 10 samples from industrial composting plants and compost reactors located in Finland, Sweden and Norway [34,35]. The LDR experiments were conducted with 10 ng of the extracted genomic DNA as a template and also with PCR fragments pooled from three replicate reactions in two different quantities, 4 ng and 20 ng. Genomic DNA (10 ng) was found to function as a template in LDR in 3 of the ten samples (NK07, NK10 and NK14B), but no signal was detected in eight samples (Table 3). After PCR amplification of the ITS-area signal was detected from several probes with both 4 ng and 20 ng of template. The signals in 20 ng were substantially higher and in some cases, not all probes, that gave positive signal in 20 ng, were among the positives in 4 ng (Table 3). The ligation and hybridisation with 20 ng of PCR product as a template was performed in three replicates. A probe was considered positive when positive signal was detected in two out of three replicates. The highest number of positive spots was detected with 20 ng of PCR product used as a template in LDR.

**Table 3:** Phylotypes detected by LDR versus results from cloning and sequencing.

	A26	A29	A45	A50	A52	A53	A54	A55	A56	A57	A58	A65	A66	A67	A83	A84	A85	A86
NK19 clone		**X**						**X**										
NK19 20 ng		**X**						**X**										
NK19 4 ng		**X**						**X**										
NK19 genomic																		

NK14b clone					**X**			**X**				**X**	**X**					
NK14b 20 ng		**X**			**X**		**X**	**X**			**X**	**X**	**X**				**X**	
NK14b 4 ng					**X**			**X**				**X**	**X**					
14b genomic											**X**		**X**		**X**			

NK14a clone					**X**			**X**				**X**	**X**					
NK14a 20 ng		**X**			**X**		**X**	**X**				**X**	**X**				**X**	
NK14a 4 ng		**X**			**X**			**X**				**X**	**X**					
NK14A genomic	**X**																	

NK12 clone					**X**		**X**	**X**					**X**				**X**	
NK12 20 ng					**X**		**X**	**X**					**X**				**X**	
NK12 4 ng					**X**		**X**	**X**					**X**					
NK12 genomic																		

NK10 clone			**X**		**X**										**X**	**X**		
NK10 20 ng			**X**		**X**													
NK10 4 ng			**X**		**X**													
NK10 genomic				**X**	**X**										**X**			

NK09 clone					**X**										**X**			
NK09 20 ng					**X**													
NK09 4 ng					**X**										**X**			
NK09 genomic													**X**					

NK8 20 ng	**X**	**X**		**X**		**X**												
NK8 4ng		**X**					**X**											
NK8 genomic																		

NK7 clone			**X**		**X**													
NK7 20 ng	**X**		**X**		**X**													
NK07 4 ng			**X**															
NK07 genomic	**X**		**X**		**X**													

NK06 clone			**X**		**X**													
NK06 20 ng			**X**		**X**													
NK06 4 ng			**X**		**X**													
NK06 genomic										**X**								

NK5 20 ng					**X**										**X**			
NK5 4 ng					**X**													
NK5 genomic																		

Different concentration of DNA was used in hybridisation (y-axis). Hybridisation with 20 ng of PCR amplified internal transcribed spacer (ITS) area DNA was done in triplicate and when two out of three replicates were positive, the phylotype was marked present. 4 ng was hybridised once. In addition, 10 mg of genomic DNA from environmental sample was used. All of the probes (x-axis) were used in the hybridisation. For phylotype information corresponding to each zip-code, see table 1.

Since microarray provides information from the phylotypes there is a probe for, clone libraries were constructed from eight samples to determine the phylotype composition in samples and find out whether the microarray test was functioning. The total number of good quality full-length sequences by Phred score 20 was 588. Sequences were clustered into 53 phylogroups with < 1% difference and the number of phylotypes in the sequenced libraries varied between 3 and 18 (Table 2). The library coverage calculated with the Chao1 method [44] ranged from 37.9% to 85.7% and with the ACE method from 37.9% to 78.1% [45]. The total diversity was therefore undersampled. The estimated richness (Table 2) with Chao1 method ranged between 3.5 and 34.7 and with ACE between 4.0 and 40.6. The BLAST alignments revealed the fungal communities in samples to be comprised of sequences similar to ascomycetous yeasts, *Zygomycetes*, *Pezizomycotina *and to a group with no reference in the sequence databases, but which had been found previously in pilot composting drums (Hultman *et al*. 200× unpublished). The 53 phylotypes were grouped into 8 phylogroups (Table 4) by the clustering in the phylogenetic tree. The phylogroups were named Group 1 (members from the group without sequence database match), Basidiomycetes (members clustering with basidiomycota sequences), *Candida *(phylotypes clustering with some of the Candida sequences), *Pezizomycotina *(phylotypes with high similarity to the *Pezizomycotina *subphylym), *Saccharomycetaceae *1 and *Saccharomycetaceae *2 (clustered with different representatives of the *Saccharomycetaceae *family), *Dipodascaceae *(phylotypes clustered with sequences from family *Dipodasceacea*) and *Zygomycetes *(clustering with sequences from different *Zygomycota*). As the numbers of phylotypes in samples, the phylotype composition varied between different composts and the physical and chemical conditions of the samples.

**Table 4:** Amount of phylotypes in clone libraries from different samples and annotation result from Genbank.

Phylotype	Phylogroup	Closest Genbank match	Reactor NK06 day 8	Reactor NK07 day 16	Reactor NK09 day 3	Reactor NK10 day 8	IVAR^1^ NK12 1 mo.	IVAR^1^ NK14a 1 mo.	IVAR^1^ NK14b 1 mo.	YTV^1^ NK19 day 21
FM177664	Basidiomycetes	Mrakia sp.				1				
FM177671	Basidiomycetes	Coprinopsis cinerea					1			
FM177679	Candida	Candida rugosa					9			
FM177672	Candida	Candida rugosa					1			
FM177678	Dipodascaceae	Dipodascus australiensis					1			
FM177644	Group1	Uncultured soil fungus	1							
FM177645	Group1	Uncultured eukaryote	1							
FM177646	Group1	Uncultured eukaryote	1							
FM177647	Group1	Uncultured eukaryote	1							
FM177648	Group1	Uncultured eukaryote	2							
FM177650	Group1	Uncultured eukaryote	1							
FM177649	Group1	Uncultured eukaryote	1							
FM177653	Group1	Uncultured eukaryote	1	2		4				
FM177665	Group1	Uncultured eukaryote	2			8				
FM177668	Group1	Uncultured eukaryote	23			6				
FM177670	Group1	Uncultured eukaryote	6			1				
FM177666	Group1	Uncultured eukaryote	9	11		4				
FM177667	Group1	Uncultured eukaryote				6				
FM177669	Group1	Uncultured eukaryote	19			49				
FM177651	Pezizomycotina	Cephalotheca foveolata		1						
FM177696	Pezizomycotina	Penicillium radicum			1					
FM177677	Pezizomycotina	Thermomyces lanuginosus					25	61	51	71
FM177676	Pezizomycotina	Trichocomaceae sp.					1	14	18	
FM177684	Pezizomycotina	Thermomyces lanuginosus					3	3		
FM177685	Pezizomycotina	Uncultured soil fungus						1		
FM177688	Pezizomycotina	Aspergillus oryzae					1	1	3	
FM177689	Pezizomycotina	Thermomyces lanuginosus						1		
FM177691	Pezizomycotina	Thermomyces lanuginosus							1	
FM177695	Pezizomycotina	Thermomyces lanuginosus								2
FM177662	Saccharomycetales 1	Issatchenkia orientalis	1	2	1	3	8	1	12	
FM177654	Saccharomycetales 1	Saccharomycetales sp.			1					
FM177663	Saccharomycetales 1	Saccharomycetales sp.			1					
FM177655	Saccharomycetales 1	Saccharomycetaceae sp.			2					
FM177656	Saccharomycetales 1	Saccharomycetales sp.			1					
FM177661	Saccharomycetales 1	Issatchenkia orientalis			69					
FM177673	Saccharomycetales 1	Uncultured eukaryote					1			
FM177680	Saccharomycetales 1	Saccharomycetales sp.			7		1			
FM177652	Saccharomycetales 2	Candida sake		1						
FM177658	Saccharomycetales 2	Kluyveromyces marxianus			1	1				
FM177659	Saccharomycetales 2	S. cerevisiae			2					
FM177660	Saccharomycetales 2	S. cerevisiae			4					
FM177675	Saccharomycetales 2	Torulaspora delbrueckii					1			
FM177681	Saccharomycetales 2	Saccharomycetales sp.					2			
FM177690	Saccharomycetales 2	Saccharomycetales sp.						1		
FM177657	Zygomycetes	Mucor sp			1					
FM177674	Zygomycetes	Absidia corymbifera					8	11	1	
FM177682	Zygomycetes	Mucor racemosus					1			
FM177683	Zygomycetes	Absidia corymbifera					1			
FM177687	Zygomycetes	Absidia corymbifera						2		
FM177686	Zygomycetes	Absidia corymbifera					2	1		
FM177692	Zygomycetes	Absidia sp.							1	
FM177694	Zygomycetes	Absidia corymbifera							1	
FM177693	Zygomycetes	Rhizomucor miehei					2		1	

The phylotypes are marked with the EMBL nucleotide sequence accession number.

^1 ^IVAR and YTV were from full-scale facilities

### Fungal composition in samples

The design of the probes used in the LDR assay was based on sequences of common phylotypes in our previous studies (Hultman *et al*. unpublished). The probes were designed for indicator phylotypes at different phases of composting such as the mesophilic stage and the active, thermophilic stage. Compared with the sequencing results, phylotypes with high similarity (> 99%) to the LDR target sequences were positive when detected with sequencing.

#### Research reactor

By cloning and sequencing the phylotype richness in research reactor samples was highest in sample NK06 (Table 2). The phylotypes in these samples were mainly from Group 1, though in both samples there were clones similar to *I. orientalis*, and in addition in sample NK07 phylotypes similar to *Candida sake *and *Sordariomycestes*. In sample NK09 the phylotypes were from *Saccharomycetaceae *and *Pezizomycotina *origin. No representatives from Group1 were detected by cloning or LDR in sample NK09 even though in sample NK10 the group represented over 90% of the sequenced clones. Group 1 was not found to be present in the initial stages of composting (Table 3) as it was absent in sample NK05 by LDR and in sample NK08 and NK09 by LDR and LDR and cloning, respectively. In samples NK09 and NK10 the phylotype for probepair KlyMa was present in clone libraries, but not detected by LDR probes.

#### Full-scale process

The highest diversity in the clone libraries was observed in sample NK12 (ACE 40.6, Table 2.) where phylotypes similar to various *Pezizomycotina*, *Zygomycota *and *Saccharomycetaceae *(Table 4.) but no members from Group1 were identified. Samples NK14a and NK14b were diverse (S ACE 24.3 and 27.5, respectively) although the temperature was increased towards thermophilic levels. The DNA in these two samples was extracted from the same sample bag at different time points to determine the possible sampling and cloning bias. Both of the samples were used as template for clone library preparation and LDR-ligation and microarray hybridisation. The phylotype composition of the samples NK14a and NK14b were rather similar comprising of *Saccharomycetales, Pezizomycotina and Zygomycetes *and in addition sample NK14a contained phylotype similar to uncultured *Saccharomycotina *(Table 4). The phylotypes similar to *Candida *and *Dipodascaceae *were declined and not detected by clone library sequencing in these samples. However, the LDR-array detected phylotypes in sample NK14 from full-scale process that were not found with sequencing (Table 3) and by using the LDR platform we could obtain better coverage than with clone sequencing; e.g. Galge, AspFum and CanRu were positive even though not found among the clones. These results showed, that the clone library sequencing results and microarray hybridisation give similar results despite the independently done DNA extraction. In sample NK19 from the clone library sequencing three phylotypes, all similar to *Thermomyces lanuginosus*, were detected. The same phylotype was found also with the LDR-array in addition to positive signal with the probe specific for *Pezizomycotina*.

## Discussion

DNA microarrays have the potential to be useful in fast and efficient monitoring of complex microbic environment but the conventional array platforms may suffer from nonspecific background signals and low sensitivity. We have applied the ligation detection reaction (LDR) microarray method for municipal biowaste compost samples to develop a tool for monitoring the fungal species contributing to different phases of the decomposition process and to demonstrate the feasibility of LDR microarray for species-level detection from complex microbial communities. Previously, LDR has been successfully applied to detecting microbes from environmental samples [19-21]. These studies report 1–5 fmol sensitivity at 5% of total DNA using genus consensus sequences in probe design and group-selective PCR primers in template amplification. Better sensitivity has been achieved by PCR amplifying the ligation products [24,47] but this approach requires long circulazing oligonucleotide probes, nuclease treatments and PCR reaction making it somewhat more expensive and time consuming than simple LDR. Here, we have been able to improve the sensitivity of the LDR method to detect 0.1 fmol of target DNA at 0.04% level of total DNA using species-specific ligation probes. As such, the method is feasible for accurate determination of compost microbiota and serves as a basis for development of a diagnostic tool. The ability to distinguish microbes at species-level is of value in many environmental studies where certain pathogenic or key species need to be detected. The developed LRD based method differs from the diagnostic oligonucleotide microarrays. For example in the high density PhyloChip [17] the range of microbes detected is much higher as the probes are designed based on different taxonomic levels. In our system the main focus was in sensitive species level detection which was achieved. In the mentioned PhyloChip the specificity was targeted to the OTU-level (sequences with 97% similarity) [17] and the main focus was in the study of microbial diversity instead of presence or absence of certain species of phylotypes.

The ribosomal internal transcribed (ITS) spacer region was selected as the target for ligation probes since its extensive use in phylogenetics offers plenty of reference material in sequence databases for probe design. In addition, the amount of ribosomal sequences in the local database was large due to our previous sequencing projects [27,28], Hultman *et al*. unpublished) providing sequence information of the relevant target species. The probe pairs were designed for 14 species or phylotypes that were found to be abundant in the industrial composting process based on previous studies (Hultman *et al. *unpublished). Target phylotypes were selected to reflect the different stages of the composting process, the acidic initial stages and the thermophilic latter stages. A group specific probe for subphylym *Pezizomycota *was designed because the number of sequences clustering to *Pezizomycota *was low in the earlier studies and the reference data was therefore low. Probes for phylotypes similar to *Absidia *and *Emericella *were designed based on preliminary sequencing of the samples studied here. The probes were designed using rRNA sequence alignments and verified with BLAST searches. The 3' terminus of the discriminating probe was designed to contain as many nucleotides as possible differentiating it from non-target templates. However, one false positive probe signal was found; IssOr probe weakly detected *Candida rugosa *template (Fig. 4), even though the probe contained mismatches to the *Candida *template, including the 3' discriminating nucleotide. Sequencing the *Candida *template did not reveal any contamination in the sample, so this false positive signal remains largely unexplained. In the experiments, AspFum probe seems to weakly detect *I. orientalis *template but this signal is below threshold (Fig. 4).

For all tested probes, the lower limit for sensitivity in our experiments was 0.1 fmol of target PCR product comprising ca. 0.04% of total DNA present in the reaction mixture, suggesting that the relatively large amount of background DNA does not limit the ligation reaction of the probes. DNA from fungal pure cultures was used as a background DNA because in the environmental samples there is non-target DNA present that can affect the hybridisation. The level of absolute sensitivity (0.1 fmol) is higher compared to what was reported earlier for LDR [21], reaching the levels of qPCR. This could possibly be attributed to the use of a hybridisation control probe and to slightly different reaction conditions than in other studies, but it is not clear to us exactly why better sensitivity was seen in this study. However, the method used is not quantitative as the template DNA is PCR-amplified prior ligation and the amplification is not done in a quantitative manner.

The hybridisation control probe measured the per-spot variance in Zip-oligo hybridisation which helped normalizing the signal between spots. Gupta and coworkers [48] utilised a similar idea in microarray experiments where a third label was successfully used to define the spot areas and to flag the spots with low quantity or no probes printed due to array printing errors. We did not systematically assess the effect of the B3 hybridisation control probe to sensitivity, but it is likely that the effect was largest at low template concentrations where the signal was weakest. The increase in sensitivity achieved here shows the potential of LDR based microarrays in sensitive species level detection.

Increasing the number of ligation reaction cycles did not substantially affect sensitivity (Fig. 3). This could be explained by the fact that the ligation products have approximately 15–20 degrees higher melting temperature than the non-ligated probes. As the reaction proceeds, the forming ligation products could effectively occupy the target sites for probe hybridisation because at annealing temperature the ratio of hybridised to non-hybridised ligation products should be quite high. Therefore, the number of ligation products can be expected to saturate as a function of reaction cycles. It is also possible that the performance of reactants and fluorescent labels or the integrity of DNA declines in high temperatures over time.

The fungal composition of the Nordic samples determined by the clone library sequencing was similar as in our previous studies in Finnish composting plants and in a pilot drum experiment [[27], Hultman *et al. *unpublished]. The samples NK06 to NK10 were from a compost reactor [34,35] and the composition of the phylotypes is similar to the composition in the pilot drum studied by Hultman and co-workers (unpublished). In sample NK09, the fungal diversity was high and the sequences clustering to *Saccharomycetaceae *1 were found frequently. Similar sequences were found in the well functioning stages of composting process in the full-scale drums [27]. Yeast-like sequences were frequently found in the Norwegian plant (samples NK12 and NK14) together with sequences similar to *Zygomycetes *and *Pezizomycota*. Hence the yeasts can be considered to be of importance to the often acidic industrial composting process by being able to grow at low pH and therefore reduce the acidity and increase the growth of thermophilic bacteria [49]. The pH in the Scandinavian samples is often low which can hinder the decomposition, especially when the temperature is about 40°C [37,50]. In sample NK19, only three phylotypes all similar to *T. lanuginosus *were found. The bacterial diversity in the same sample was relatively high [34,35] so the reason for the low fungal diversity was not unrepresentative sample.

The results from the clone library sequencing were compared with the microarray hybridisation results. Most of the phylotypes present in clone libraries were also detected by the microarray. However, in samples NK14a and NK14b, some phylotypes not observed by clone sequencing were nonetheless detected by LDR (Table 3). Sequence dependent cloning bias might have affected the representativeness of the library (reviewed in [51]) and it is conceivable that some fragments were left out from cloning. On the other hand, *Kluyveromyces marxianus *phylotype was present in the clone library but was not properly detected by LDR. In sample NK10, *K. marxianus *was detected with LDR from the genomic DNA as a template but not with the PCR-product as a template. In NK09, *K. marxianus *probe was positive with 4 ng of PCR-product used as a template but not with 20 ng of template. The number of sequenced clones similar to *K. marxianus *was only one in both libraries NK09 and NK10, which suggests that the number of template fragments amplified in PCR was in fact too low to be over the detection threshold.

## Conclusion

The probes detected indicator phylotypes for different phases of composting such as the mesophilic stage and the active, thermophilic stage. The microarray detection limit was 0.04% of the total DNA showing potential for use of the method for example in pathogen detection. The microarray was found to be effective in detecting certain phylotypes when applied to complex environmental samples taken from the early stages of municipal biowaste composting. The microarray results were succesfully verified by sequencing of clone libraries. Ongoing studies will widen the probe pool and expand it to include the bacteria. The plattform will then serve as a basis for a diagnostic environmental monitoring tool.

## Authors' contributions

JH participated in the design of the study and coordinated the clone library preparation, did the microarray experiments, analysed the data and drafted the manuscript. JR wrote the R-script for the microarray analysis, did the microarray experiments, analysed the data and drafted the manuscript. MR coordinated the sampling and participated in writing the manuscript. LP participated in the design of the study. PA coordinated the study and contributed in writing the manuscript. All authors read and approved the final manuscript.
